# Association between Children's Physical Activity and Parental Practices Enhancing Children's Physical Activity: The Moderating Effects of Children's BMI z-Score

**DOI:** 10.3389/fpsyg.2017.02359

**Published:** 2018-01-25

**Authors:** Natalia Liszewska, Urte Scholz, Theda Radtke, Karolina Horodyska, Michal Liszewski, Aleksandra Luszczynska

**Affiliations:** ^1^Wroclaw Faculty of Psychology, SWPS University of Social Sciences and Humanities, Wroclaw, Poland; ^2^Department of Psychology, University of Zurich, Zurich, Switzerland; ^3^Trauma, Health, and Hazards Center, University of Colorado at Colorado Springs, Colorado Springs, CO, United States

**Keywords:** children, physical activity, parental practices, body mass, BMI

## Abstract

**Objective:** Parental practices that aim at increasing children's physical activity were found to be related to children's physical activity. So far, however, the association between these two sets of variables was studied without considering the moderating role of children's BMI z-score, which may determine the effectiveness of parental practices. The present study aims at filling this void.

**Design:** Longitudinal data were collected among 879 dyads of children (6–11 years old) and their parents. Seven parental physical activity practices were assessed at baseline. Physical activity, body mass, and height (measured among children) were assessed twice (at baseline and 7-month follow-up). Body mass and height were measured objectively. Seven moderation analyses were conducted.

**Results:** Six parental practices emerged to predict physical activity of children: collaborative social control, overall support, stimulation to be active, general encouragement for physical activity, positive social control, and modeling. Children's BMI z-score moderated three associations. The relationships between parental positive social control, overall parental support, and general parental encouragement for physical activity (at baseline), and children's physical activity (at follow-up) were significant only among children with low and medium BMI z-score. In turn, collaborative social control and modeling predicted children's physical activity at the follow-up regardless child's BMI z-score.

**Conclusions:** Parental positive social control or overall parental support may be ineffective in children with higher body mass who are in need to increase their physical activity.

## Introduction

The occurrence of childhood obesity is a continuously developing trend (Wild et al., 2004; Reilly, 2006). Obesity among children may result in health, emotional, and social problems (Dietz, 1998; Schwimmer et al., 2003). Consequently, one of the key tasks of health and social sciences is to identify any modifiable practices or strategies, which can help to prevent obesity or constitute risk, or causal factors of obesity among children (Cachelin et al., 2014).

One of the factors related to overweight and obesity is a lack of physical activity (Metcalf et al., 2008), in particular, irregularity and insufficient intensity of physical activity (Stallmann-Jorgensen et al., 2007). Physical activity is crucial for the healthy growth of children and adolescents and it helps to prevent chronic illnesses later in life (US Department of Health Human Services, 1996; World Health Organization, 2008). Studies conducted among children showed that the relationship between the BMI z-score and the regularity of physical activity is inverse (Haerens et al., 2010).

Parental behavior may have a considerable influence on children's BMI and physical activity (Golan and Crow, 2004; Gubbels et al., 2011; Sleddens et al., 2011). Parental practices are treated as content-specific acts of parenting (Darling and Steinberg, 1993), such as rules of activity behavior. The role of parental behavior for the development of children's physical activity is specified in several behavior change theories, including social cognitive theory (Bandura, 2001) and the theory of planned behavior (Ajzen, 2002). Due to modeling the behavior of people important to an individual has influence on behavior and beliefs formulation of that person (Bandura, 2001). According to the theory of planned behavior normative beliefs, which refer to approval of important people, determine behaviors (Ajzen, 2002).

Initiation and maintenance of regular physical activity originate in early childhood (Reilly et al., 2004), and track into later life (Sallis et al., 1992b; Kelder et al., 1994). Parents may foster children's engagement in physical activity using a variety of mechanisms, such as exercising together with their children, verbally encouraging children's engagement in physical activity (Edwardson and Gorely, 2010), and by applying particular parental practices (Golan and Crow, 2004).

So far, research on parental practices influencing child's physical activity identified parental social support and modeling as important factors operating in children's and adolescents' physical activity (Sallis et al., 2000; Strauss et al., 2001; Davison and Schmalz, 2006; Gustafson and Rhodes, 2006; Springer et al., 2006). However, as reported in a review by Ferreira et al. (2007), there are also some studies failing to find an effect of these practices on children's physical activity. Cross-sectional research on preschool and school children (age 3–11) and their parents showed that parents who are physically active are more likely to support their children's engagement in physical activity (Klesges et al., 1990; Welk et al., 2003). Parental assistance in physical activity (including transporting the child to various exercises or sporting venues, or providing equipment, access or opportunities to be active) was strongly connected with children's physical activity levels (Sallis et al., 1992a; Welk et al., 2003). Overall, these inconclusive research results indicate an important gap in the literature as it is still unresolved under what conditions modeling and parental social support are effective for children's physical activity.

Another strategy of parental behavior to promote children's physical activity is social control. It has also been reported to be a relevant parental practice applicable for children 6–12 years old (Wilson et al., 2007, 2010). Social control can be expressed in multiple forms. Two main types of social control strategies have been named: positive and negative (Lewis and Butterfield, 2005; Fekete et al., 2006). Positive strategies are supposed to encourage children's physical activity, and may involve discussion or prompting, whereas negative ones are supposed to put pressure to children's physical activity, and may involve the use of sense of guilt or disapproval. Further research (Wilson et al., 2007) revealed the need for defining a third type of control, that is collaborative social control, which is requiring action on both sides, parent and child (e.g., the parent suggesting joint activities with the child). Positive and collaborative social control, but not negative control have also been reported to be related to children's activity following a children's activity lapse. For example a relationship between children's activity and positive and collaborative social control was identified, but there was no relationship with negative social control (Wilson et al., 2010). A lapse is understood in that case as a short period of time with very little or a lack of activity (Conroy et al., 2007).

Across weight-related behaviors, systematic approaches and taxonomies of parental practices influencing child's BMI were proposed only for nutrition-related practices (de Lauzon-Guillain et al., 2012). Unfortunately, studies on parental influences on physical activity are less systematic and researchers often select only certain types of practices (e.g., Gustafson and Rhodes, 2006; Edwardson and Gorely, 2010), instead of testing for the effects of a broader range of practices. The present study will address this issue, investigating the effects of seven parental strategies, including parental modeling, positive, negative, collaborative social control, overall support, stimulation to be active, and general encouragement for physical activity.

Furthermore, there is a lack of empirical evidence taking into consideration moderators of parental influences on child physical activity behavior. Similarly to diet, physical activity is a weight-related behavior (cf. Horodyska et al., 2015), hence the mechanisms between parental practices and weight related behaviors may be similar in both domains. Children's BMI is one of the moderating mechanisms for parental practices and diet (Gubbels et al., 2009). Consequently, as also recommended by Gubbels et al. (2011) and demonstrated by Taylor et al. (2002) further research is needed to examine whether parental practices and physical activity are differently related depending on children's body mass. Thus, the present study set out to close this gap by investigating if children's BMI z-score may serve as a moderator between parental practices and children's physical activity.

The goal of this study is to systematically examine a wide range of parental practices related to increase of physical activity level of children aged 6–11. In particular, we aimed at investigating the relationship between the practices used by parents (modeling, positive, negative, collaborative social control, overall support, stimulation to be active, general encouragement for physical activity), indicators of children's BMI z-score, and physical activity of children aged 6–11, controlling for the effects of age and sex of the child. A longitudinal design (with 7 months between measurement points Time 1 and Time 2) was applied, with objectively measured body weight and height. In particular, we hypothesized that parental practices measured in parents (Time 1) would predict child's physical activity at the 7-month follow-up, with children BMI z-score (Time 1) moderating this relationship. These associations were assumed to be significant while controlling physical activity at the baseline, gender, and age of the child.

## Materials and methods

### Participants: parents and children

At Time 1 (T1), 879 parents and children participated in the study; 603 of them completed the measurement at **7-**month follow-up (Time 2, T2). Children (Ch) were 6–11 years old (*M* = 8.46, *SD* = 1.34); 47.6% of them were boys. According to WHO cut-offs (de Onis et al., 2007) 29.6% of children were overweight or obese, adjusting to age and gender.

The parents who participated in the study were 24–68 years old (*M* = 36.67, *SD* = 6.09), 82.9% were women. Most of the parents had either at least a bachelor's degree (40%), or secondary education (40.5%), while 19.5% had primary education only. The research was performed in rural areas (29.8%), towns up to 10,000 citizens (26.2%) and bigger cites (44%) in Poland. The study was approved by the Internal Review Board at the first author's institution.

### Procedure

The two measurement points were separated by a 7 months interval. The 13 experimenters (all with a MA degree in psychology) participated in a comprehensive research procedure training. Children and parents were given information about the research schedule a week before the study began and were asked to confirm participation and provide their informed consent (parent's provided consent for children's participation). Participants were informed about the aims of the study and ensured about the anonymity by experimenters.

Data from children were collected with standardized individual interviews and questionnaires (paper-pencil method). The data collection lasted between 15 and 30 min depending on children's age and reading/writing skills. In case of younger children, the experimenters read the question and gathered oral answers from them. After the questionnaire had been completed, the body weight and height were measured with the usage of the certified body weight scales (all used devices had ISO certification and the measuring device error below 5%). The children's data were collected in primary schools (school nurse's offices), kindergartens, and at participants' homes. Parents were asked to fill in the questionnaires. They supplied their data at their children's school, kindergarten, or at home.

In order to conduct the T2 measurement, the experimenters revisited the schools 7 months later after contacting parents by phone. The attempt to contact parents was repeated three to five times during a 3-week period of time so parents who were temporarily absent were able to participate the second measurement time.

### Measurement

#### Measures applied in parents

Parental practices (measured among parents) referred to seven types of practices related to children's physical activity. Across the measures, parents were asked to respond on a scale from 1 (definitely not) to 4 (exactly true).

Stimulation to be active was measured with three items derived from Activity-related Parenting Questionnaire by Gubbels et al. (2011). The examples of items were “If my child says, ‘I don’t feel like walking or bicycling' I try to get him/her to do this anyway.” Descriptive statistics at T1 were *M* = 3.07, *SD* = 0.68. The reliability of the scale was considered to be good at T1 (α = 0.77).

Modeling of physical activity was measured with four items. We changed the original modeling diet-related items of the Comprehensive Feeding Practices Questionnaire developed by Musher-Eizenman and Holub (2007) so they would represent the context of physical activity. The examples of items were: “I can model physical activity of my child by engaging in physical activity on my own.” The Cronbach's alpha coefficient was 0.86 (T1). The descriptive statistics at T1 were *M* = 2.58, *SD* = 0.73.

Positive social control (SC) and the other two types of social control (negative SC, collaborative SC) identified previously in the research with child-parent dyads (Wilson et al., 2007, 2010), were used to assess social control. An example item is “How likely is it that you would encourage your child to stick with his/her physical activity?” The descriptive statistics at T1 were *M* = 3.02, *SD* = 0.63. The reliability of the scale was considered to be good at T1 (α = 0.74).

Negative social control was measured with two items. An example item is “How likely is it that you would nag your child to be active as a means of increasing your child's physical activity?” (Wilson et al., 2007, 2010). The descriptive statistics at T1 were *M* = 2.21, *SD* = 0.74. The Pearson correlation coefficient between items at T1 was *r* = 0.62, *p* < 0.001.

Collaborative social control was assessed with three items. An example item is “How likely is it that you would participate in physical activity so the child could see it as a means of increasing your child's physical activity?” (Wilson et al., 2007, 2010). The descriptive statistics at T1 were *M* = 2.74, *SD* = 0.63. The reliability of the scale was considered to be good at T1 (α = 0.68).

Overall support provided by parents, mother or father, was measured with five items. Different types of support were measured, for instance: encouragement, transport, attitudes, organization, supervision. An example item is “I take my child to the places where they can play sports” (Edwardson and Gorely, 2010). The reliability of the scale was considered to be good at T1 (α = 0.84). The descriptive statistics at T1 were *M* = 3.11, *SD* = 0.69.

General encouragement to be physically active was measured with 21 items. The additional index was based on the mean item scores from the six scales mentioned above (all scales had the same response format). An exploratory factor analysis resulted in one factor accounting for 56% of the total variance, which justifies combining six scales to one general index of physical activity enhancement (eigenvalue = 3.36). The descriptive statistics at T1 were *M* = 2.91, *SD* = 0.53. The reliability of the scale was considered to be good at T1 (α = 0.92).

#### Measures applied in children

Children's physical activity was measured at T1 and T2 with a validated self-report by Godin and Shephard (1985). Participants were told to report any exercise session that lasted at least 15 min (one bout), conducted outside of the physical education class at school, and which was not associated with organized sport activities. The obtained physical activity index accounts for bouts of physical activity and the metabolic values of physical activity per week. The index is calculated with a formula: total exercise score = 9 × [strenuous bouts per week] + 5 × [moderate bouts per week] + 3 × [mild bouts per week] (Godin and Shephard, 1985). The descriptive statistics were *M* = 55.35, *SD* = 31.43, minimum 0.00, maximum 313.00 (T1) and *M* = 57.60, *SD* = 28.19, minimum 0.00, maximum 281.00 (T2).

Children's body mass index was calculated with the data of body weight, height, age, and gender of children, which were collected at T1. A certified body weight scale was used to measure body weight (Beurer; measurement error <5%). BMI z-scores were calculated with WHO growth references by using SPSS macro provided by WHO (de Onis et al., 2007). The descriptive statistics were *M* = 0.44, *SD* = 1.24, minimum −4.82, maximum 4.41 (T1). The values for the moderator BMI z-score were: low (minus one *SD* from mean, *M* = −0.80), the mean (*M* = 0.44) and high (plus one SD from mean, *M* = 1.68).

### Data analysis

The hypotheses were tested by means of regression analysis with 10,000 bootstrapping, using the PROCESS macro (model 1; Hayes, 2013). We complied with Hayes's guidelines (2013) and demonstrated the model significance by following the approach of bootstrap confidence intervals. Thus, we do not report the *p*-value for the specific paths, as that would mean agreeing to the regular theory approach. Instead we determined unstandardized coefficient value for each of the paths. We used bias-corrected confidence intervals with 10,000 bootstrap samples.

A total of seven moderation analyses were conducted, resulting from testing the relationships between seven kinds of parental practices and the dependent variable, physical activity of children, taking into consideration the BMI z-score as the moderator. Moreover, the following covariates were entered in the analyses in order to account for their potential influence on physical activity of children at the follow-up: physical activity measured at T1, children's age and gender. The three cut-off values for the moderator were: low (minus one *SD* from mean, *M* = −0.80), the mean (*M* = 0.44) and high (plus one *SD* from mean, *M* = 1.68).

Missing data for all variables were replaced using the multiple imputation method (Schafer and Graham, 2002; Streiner, 2002).

## Results

### Preliminary analyses

Dropout analyses demonstrated that there was no significant difference between the parents who dropped out and those who remained in terms of age *F*_(1, 868)_ = 2.77, *p* = 0.10, BMI *F*_(1, 862)_ = 0.09, *p* = 0.77, gender $\chi_{\left(1,\;874\right)}^{2}$ = 0.88, *p* = 0.38, and parental physical activity practices: stimulation to be active *F*_(1, 876)_ = 1.09, *p* = 0.30, collaborative social control *F*_(1, 874)_ = 0.06, *p* = 0.81, negative social control *F*_(1, 871)_ = 1.60, *p* = 0.21, positive social control *F*_(1, 875)_ = 0.002, *p* = 0.97, modeling *F*_(1, 874)_ = 0.07, *p* = 0.80, overall support *F*_(1, 875)_ = 0.000, *p* = 0.98, general encouragement for physical activity *F*_(1, 876)_ = 0.14, *p* = 0.71. Among children who did not participate in both measurement points and those who did there were no differences in terms of gender, $\chi_{\left(1,\;874\right)}^{2}$ = 0.36, *p* = 0.38, and physical activity at T1 *F*_(1, 877)_ = 3.66, *p* = 0.06. However, there was a systematic difference in the BMI z-score at T1, *F*_(1, 862)_ = 9.76, *p* = 0.01. Dyads with children with higher BMI z-score more often resigned from participation in the second measurement.

Results of correlation analyses and descriptive statistics for the total sample (*N* = 879) are presented in Table 1. Parental practices such as stimulation to be active, positive social control, overall support, and general encouragement for physical activity reported by parents were associated with higher level of children's physical activity (T1 and T2). Moreover, negative associations emerged between children's BMI z-score (T1) and parents' overall support. There was no significant relationship between children's BMI z-score and children's physical activity at (T1), but there was a significant relationship at (T2). Also, parental age and gender were unrelated to children's physical activity (T2); children's gender was associated with children's physical activity (T2).

**Table 1:** Correlations between study variables.

	**Variable**	**1**	**2**	**3**	**4**	**5**	**6**	**7**	**8**	**9**	**10**	**11**	**12**	**13**
1	Physical activity (Ch; T1)													
2	Physical activity (Ch; T2)	0.290^**^												
3	BMI z-score (Ch; T1)	−0.010	0.076^*^											
4	Stimulation to be active (P; T1)	0.120^**^	0.117^**^	−0.004										
5	Collaborative social control (P; T1)	0.067^*^	0.100^**^	−0.058	0.449^**^									
6	Negative social control (P; T1)	0.059	0.055	0.060	0.387^**^	0.343^**^								
7	Positive social control (P; T1)	0.227^**^	0.153^**^	0.020	0.270^**^	0.294^**^	0.114^**^							
8	Modeling (P; T1)	0.038	0.122^**^	−0.027	0.336^**^	0.699^**^	0.304^**^	0.210^**^						
9	Overall support (P; T1)	0.148^**^	0.141^**^	−0.070^*^	0.507^**^	0.491^**^	0.269^**^	0.379^**^	0.453^**^					
10	General encouragement (P; T1)	0.130^**^	0.145^**^	−0.042	0.688^**^	0.791^**^	0.534^**^	0.383^**^	0.769^**^	0.806^**^				
11	Sex (P; T1)	0.016	−0.030	0.003	0.064	−0.068^*^	−0.079^*^	0.022	−0.119^**^	−0.029	−0.061			
12	Sex (Ch; T1)	−0.113^**^	−0.073^*^	−0.043	−0.083^*^	−0.024	−0.073^*^	−0.052	−0.022	−0.145^**^	−0.102^**^	0.028		
13	Age (P; T1)	0.009	0.045	−0.106^**^	0.048	0.014	0.050	0.047	0.056	0.179^**^	0.113^**^	−0.194^**^	−0.001	
14	Age (Ch; T1)	0.050	0.064	0.085^*^	−0.054	−0.071^*^	0.021	0.045	−0.060	0.070^*^	−0.003	−0.079^*^	−0.010	0.184^**^

* p < 0.05;

** *p < 0.01; Ch, the variable was measured in children; P, the variable was measured in parents; T1, Time 1; T2, Time 2 (7-month follow-up)*.

### Effects of parental practices on children physical activity moderated by children's BMI z-score

Our hypothesis suggested that child's BMI z-score (Ch; T1) would moderate the relationship between parental practices used to enhance children's physical activity (P; T1) and reported physical activity of children (Ch; T2). The analyses controlled for children's age, gender, and physical activity at Time 1 (Ch; T1).

First, direct effects of parental practices on physical activity were analyzed. Significant relationships between parental practices associated with physical activity (P; T1) and physical activity of children (Ch; T2) were found for six practices: collaborative social control, overall support, stimulation to be active, general encouragement for physical activity, modeling, and positive social control (see Table 2). Overall, a more frequent use of these practices was associated with a higher level of children's physical activity. The main effect of negative social control on children's physical activity was not significant.

**Table 2:** Effect of parental physical activity practices (T1) on children's physical activity (T2) moderated by child's BMI z-score.

	**Direct effect on physical activity (Ch; T2)**				**Interaction effect of BMI z-score (the moderator) and parental practices (P; T1)**					
	***B***	***SE***	**95% CI**	**CI**	***B***	***SE***	***R^2^***	**Δ*R^2^***	**95% CI**	**CI**
1. Positive social control (P; T1)	**3.89**	**1.39**	**1.17**	**6.62**	–**0.91**	**1.04**	**0.10**	**0.001**	–**2.96**	–**1.14**
Gender (Ch, T1)	−1.94	1.82	−5.51	1.64						
Age (Ch, T1)	0.88	0.68	−0.45	2.22						
Physical activity (Ch, T1)	**0.25**	**0.03**	**0.18**	**0.30**						
2. Negative social control (P; T1)	1.58	1.30	−0.97	4.14	−0.97	0.96	0.09	0.001	−2.86	0.92
Gender (Ch, T1)	−1.95	1.83	−5.55	1.65						
Age (Ch, T1)	0.92	0.68	−0.41	2.26						
Physical activity (Ch, T1)	**0.26**	**0.03**	**0.20**	**0.32**						
3. Collaborative social control (P; T1)	**4.60**	**1.51**	**1.63**	**7.55**	−1.39	1.03	0.10	0.002	−3.42	0.64
Gender (Ch, T1)	−1.87	1.82	−5.45	1.71						
Age (Ch, T1)	1.05	0.68	−0.28	2.38						
Physical activity (Ch, T1)	**0.26**	**0.03**	**0.19**	**0.32**						
4. Stimulation to be active (P; T1)	**4.38**	**1.53**	**1.39**	**7.36**	−1.41	1.12	0.10	0.002	−3.61	0.79
Gender (Ch, T1)	−1.62	1.83	−5.21	1.98						
Age (Ch, T1)	1.03	0.68	−0.30	2.37						
Physical activity (Ch, T1)	**0.25**	**0.03**	**0.19**	**0.32**						
5. Modeling (P; T1)	**5.14**	**1.28**	**1.26**	**10.90**	−1.72	0.93	0.11	0.004	−3.53	0.10
Gender (Ch, T1)	−1.81	1.82	−5.38	1.76						
Age (Ch, T1)	1.10	0.68	−0.23	2.43						
Physical activity (Ch, T1)	**0.26**	**0.03**	**0.20**	**0.32**						
6. Overall support (P; T1)	**5.30**	**1.42**	**2.51**	**8.10**	–**2.34**	**1.02**	**0.11**	**0.005**	–**4.35**	–**0.33**
Gender (Ch, T1)	−1.09	1.84	−4.70	2.51						
Age (Ch, T1)	0.78	0.68	−0.55	2.11						
Physical activity (Ch, T1)	**0.25**	**0.03**	**0.19**	**0.31**						
7. General encouragement (P; T1)	**7.69**	**1.90**	**3.96**	**11.42**	–**3.04**	**1.28**	**0.11**	**0.006**	–**5.55**	–**0.52**
Gender (Ch, T1)	−1.23	1.83	−4.81	2.36						
Age (Ch, T1)	0.97	0.68	−0.35	2.30						
Physical activity (Ch, T1)	**0.25**	**0.03**	**0.19**	**0.31**						

*P, the variable measured in parents; Ch, the variable measured in children; T1, Time 1; T2, Time 2. Significant interactions are marked in bold. In all analyses, child's physical activity (T1), gender and age were controlled*.

Second, we investigated the moderating effects of children's BMI z-score. A significant interaction effect between the BMI z-score and parental practices associated with physical activity was shown with the child's level of physical activity as a dependent variable for three practices: positive social control, overall support, general encouragement for physical activity (see Table 2). There were no significant interaction effects between collaborative social control, negative social control, modeling, stimulation to be active and children's BMI z-scores on T2 physical activity.

Further analyses of the significant interaction effects revealed that higher levels of positive social control (T1) were related to a high level of physical activity (T2) only among children with a low and medium BMI z-score. At high values of the BMI z-score the relationship between parents' positive social control (T1) and children's physical activity (T2) was insignificant (see Table 3).

**Table 3:** Effect of parental practices associated with physical activity as independent variable on physical activity of children as dependent variable and the BMI z-score as moderator.

**Direct effect of parental practices on children's physical activity**	**Physical activity (Ch; T2)**											
	**Low BMI z-score**				**Medium BMI z-score**				**High BMI z-score**			
	***B***	***SE***	**95%*CI (low)***	**95%*CI (high)***	***B***	***SE***	**95%*CI (low)***	**95%*CI (high)***	***B***	***SE***	**95%*CI (low)***	**95%*CI (high)***
Positive social control (P; T1)	**4.63**	**1.83**	**1.03**	**8.23**	**3.39**	**1.32**	**0.91**	**6.08**	2.36	1.87	−1.31	6.02
Negative social control (P; T1)	2.36	1.72	−1.00	5.73	1.15	1.23	−1.26	3.57	−0.06	1.72	−3.43	3.31
Collaborative social control (P; T1)	**5.71**	**1.96**	**1.88**	**9.55**	**3.98**	**1.42**	**1.20**	**6.76**	2.24	1.87	−1.43	5.92
Stimulation to be active (P; T1)	**5.51**	**2.03**	**1.52**	**9.50**	**3.76**	**1.42**	**0.97**	**6.53**	2.00	1.94	−1.80	5.80
Modeling (P; T1)	**6.52**	**1.67**	**3.24**	**9.80**	**4.38**	**1.21**	**2.00**	**6.76**	2.24	1.67	−1.04	5.52
Overall support (P; T1)	**7.18**	**1.90**	**3.46**	**10.90**	**4.27**	**1.32**	**1.67**	**6.86**	1.36	1.77	−2.12	4.84
General encouragement (P; T1)	**10.13**	**2.24**	**5.28**	**14.97**	**6.35**	**1.77**	**2.87**	**9.83**	2.58	2.30	−1.94	7.09

*P, the variable measured in parents; Ch, the variable measured in children; T1, Time 1; T2, Time 2. Significant interactions are marked in bold*.

With regard to the interaction between overall parental support and BMI z-scores, the higher levels of parental overall support (T1) were related to a high level of physical activity (T2) only among children with low and medium BMI z-score. At high values of BMI z-score the association between the overall support (T1) and physical activity (T2) was insignificant (see Table 3).

Finally, the higher levels of parental general encouragement for physical activity (T1) were related to a high level of physical activity (T2) only among children with low and medium BMI z-score. At higher BMI z-scores the relationship between children's physical activity (T2) and general encouragement for physical activity (a combined index of physical activity enhancement) from parents (T1) was not significant (see Table 3).

## Discussion

The present study provides an insight into relationships between parental practices to enhance their children's physical activity, children's physical activity, and children's BMI z-score. Six parental practices (P; T1) emerged to be especially relevant for physical activity of children (Ch; T2): collaborative social control, overall support, stimulation to be active, general encouragement for physical activity, positive social control, and modeling. Further, children's BMI z-score made a difference with regard to the relationship between children's physical activity and parental positive social control, overall support, and general encouragement for physical activity. The associations between these three parental practices and children's physical activity were significant mostly among children with lower and medium BMI z-score, whereas the associations between any parental physical activity practices (P; T1) and children's physical activity (Ch; T2) were insignificant among children with higher levels of BMI z-score. Thus, among children with low BMI z-score the more often parents were using three respective practices the more likely their children's were physically active.

Results of research are consistent with social cognitive theory and show that various behaviors of parents, who are of key relevance to young children, influence children's physical activity behavior and consequently their body weight (Bandura, 2001). Importantly, these effects are salient among children with low and medium BMI z-scores. Future studies should include the indicators of parental body mass in order to verify whether parental body mass interacts with the effectiveness of parental practices.

Research results are also in line with ecological model of predictors of childhood overweight (Davison and Birch, 2001). According to this model parents play a crucial role in shaping children's dietary, activity and sedentary behavior. It also highlights the influence of children's characteristics, such as age, gender, and weight status on selection of parenting practices. In other words the interactions between the characteristics and behaviors of parent and the child are considered bi-directional.

Children's BMI z-score was a significant moderator of the effects of specific parental practices, namely positive social control, overall support, and general encouragement for physical activity. It is consistent with the results of current research on positive social control (Wilson et al., 2010), overall support (Gustafson and Rhodes, 2006), and general encouragement for physical activity (Sallis et al., 2000). However, existing studies did not take BMI as moderator into account. By doing so, our research went beyond the previous findings and highlighted the relevance of children's BMI z-score for the associations between parental practices and children's physical activity across 7 months. Furthermore, this study verifies these relationships by using reports from both children and their parents and by using an objective measure of BMI z-score.

There are several studies which show that parents introduce different types of activities related to food restriction if children are at risk for developing overweight or obesity (Birch and Fisher, 2000; Francis et al., 2001). Our study shows a different pattern of relationships, namely that some parental practices may be unrelated to children's physical activity if a child has a high body mass. Future studies need to explain whether parents of overweight children do not use such techniques, or whether children with higher levels of BMI z-score do not react to such parental practices.

Similarly to other research, our study did not find any evidence for relationships between child's gender and any of parental practices or the level of physical activity of the child (Salmon et al., 2005; Gubbels et al., 2011). In contrast, children's age was a relevant variable in the analyses of child's physical activity and the following parental practices: modeling, stimulation to be active, collaborative social control, and general encouragement for physical activity.

Besides many strengths of this study, such as the objective measurement of body weight, a longitudinal design, a large sample size, and dyadic data from parent-children dyads, the present study also has several limitations. The need of social approval, which could have affected parental and children's self-reports was not controlled for. Physical activity of children was measured with self-report measures; future research should incorporate objective methods of measuring physical activity, such as accelerometers.

Regardless of the limitations, this study provides important evidence for explaining the patterns of relationship between parental practices and children's physical activity. Especially, for children with lower BMI, parental use of positive social control, overall support, and general encouragement for physical activity was positively related to children's levels of physical activity. This opens up important questions regarding effectiveness of interventions to reduce childhood obesity.

While preparing interventions practitioners should consider the fact that the same parents' behavior designed to promote physical activity can be effective depending on the level of obesity of children. Future experimental research need to explore effectiveness of parental practices engaging in physical activity of obese children.

In conclusion, several parental practices emerged to be relevant for physical activity of children. In particular, we observed direct effects of collaborative social control, overall support, stimulation to be active, general encouragement for physical activity, positive social control, and modeling. Children's BMI z-score was a significant moderator of the effects of specific parental practices, namely positive social control, overall support, general encouragement for physical activity. Thus three relationships were significant among children with low and medium BMI z-score only. In turn, collaborative social control and modeling predicted children's physical activity at the follow-up regardless child's BMI z-score. In turn, the main effect of negative social control on children's physical activity was not significant.

## Author contributions

NL, US, TR, KH, AL: contributed to the conception and design of the work, analysis, and interpretation of the data. NL, KH: contributed to the acquisition of data. All authors contributed to drafting the work or revising it critically for important intellectual content and approved the final version submitted. All authors agreed to be accountable for all aspects of the work in ensuring that questions related to the accuracy or integrity of any part of the work are appropriately investigated and resolved.

### Conflict of interest statement

The authors declare that the research was conducted in the absence of any commercial or financial relationships that could be construed as a potential conflict of interest.
